# Public perception and community-level impact of national action plans on antimicrobial resistance in Vietnam

**DOI:** 10.1093/jacamr/dlad146

**Published:** 2023-12-28

**Authors:** Shruthi Anna Thomas, Philip Mathew, Jaya Ranjalkar, Thi Bich Van Nguyen, Vu Thi Quynh Giao, Sujith J Chandy

**Affiliations:** ReAct Asia Pacific, Christian Medical College, Vellore, Tamil Nadu, India; ReAct Asia Pacific, Christian Medical College, Vellore, Tamil Nadu, India; ReAct Asia Pacific, Christian Medical College, Vellore, Tamil Nadu, India; Ausvet Representative Office, Ho Chi Minh City, Vietnam; Oxford University Clinical Research Unit, Hanoi, Vietnam; Department of Pharmacology & Clinical Pharmacology, Christian Medical College, Vellore, Tamil Nadu, India

## Abstract

**Objectives:**

Vietnam was the first country from the WHO Western Pacific Region to adopt a national action plan (NAP) on antimicrobial resistance (AMR) in 2013. The multilayered nature of AMR requires coordination across ‘One Health’ sectors, dedicated financing, multistakeholder involvement, and widespread community engagement to implement the action plans. This study explores the perceived impact of NAP implementation at the community level.

**Methods:**

Key informant interviews (KIIs) were used for data collection during 2021. An interview tool was used for the KIIs and purposive sampling was used to identify study participants from Vietnam. The study participants were those engaged with a substantial scale of antimicrobial usage, diagnosis of infections or concerned with antimicrobial content in effluents in their professional life. Twelve KIIs were conducted with participants from human health, animal health and the environmental sector. The data were entered into Microsoft Excel, and manifest and latent content analysis was done.

**Results:**

The analysis highlighted themes such as limited public awareness of AMR, ongoing capacity building and quality assurance initiatives, implementation of guidelines and regulations for AMR containment, sustained investment in improving infrastructure, and challenges relating to accountability whilst prescribing and selling antibiotics.

**Conclusions:**

There were many positive critical developments during the NAP implementation period in Vietnam towards AMR mitigation. For better impact, there is a need to revitalize the implementation machinery of NAPs by improving the enforcement capacity of regulations, cross-sectoral collaboration and promoting community ownership.

## Introduction

### Background

Antimicrobial resistance (AMR) is a multifaceted problem with enormous consequences for individuals, healthcare systems and the attainment of sustainable development goals.^1^ It has been recognized as a global health threat with the potential to disproportionately impact low-resource settings.^2^

As per latest estimates, 4.95 million deaths in 2019 were associated with bacterial resistance alone and 1.27 million deaths were attributable to the same.^2^ Furthermore, the economic costs of AMR are huge, as the World Bank estimates annual global GDP to reduce by 3.8% and 1.1% by the year 2050 in high-impact and low-impact scenarios, respectively, thus pushing millions of households into poverty.^3^

The WHO, along with the Food and Agriculture Organization (FAO) of the United Nations, and the World Organisation for Animal Health (WOAH), published the Global Action Plan (GAP) on AMR in 2015 during the 68th World Health Assembly.^4^ Currently, many countries all over the world have their own national action plans (NAPs) on AMR, each at their own stage of progress.^5^

### Roadmap to NAPs on AMR in Vietnam

Vietnam is an independent middle-income country in the Southeast Asia (SEA) region with a population of 98 168 829 and an annual GDP growth rate of 2.6% in 2021. The country has a higher universal health coverage index than the regional and global averages, with 87% of the population covered.^6^

Recognizing the threat of AMR early on, the country published a situational analysis report in 2010. The report was jointly prepared by the Global Antibiotic Resistance Partnership, Vietnam National Working Group, jointly led by the National Hospital of Tropical Diseases, and the Oxford University Clinical Research Unit (OUCRU), with representation from the Vietnamese Ministry of Health (MoH) and the Ministry of Agriculture and Rural Development (MARD).^7^ Similar to other low middle-income countries (LMICs), there are challenges in regulating over-the-counter antibiotic sale in Vietnam.^8^

In 2013, the Vietnamese MoH published its NAP on AMR (2013–20) entitled ‘National Action Plan on Combating Drug Resistance’. Vietnam was the first country in the WHO Western Pacific Region to develop a NAP on AMR. The plan contained six strategic objectives and the underlying activities were split between Phase 1 (2013–16) and Phase 2 (2016–20). These have comparable priorities as outlined by the GAP, except however, with a predominant focus on human health.^9^ Vietnam’s NAP aimed to develop a national AMR surveillance system and better educate the public on antimicrobial use (AMU) and AMR.

In 2017, the ‘National Action Plan for the Management and Use of Antibiotic and Control of Antibiotic Resistance in Livestock Production and Aquaculture’ was released by MARD with five objectives.^10^ Some of the other key programmes in Vietnam supporting AMR containment are listed in Table 1.

**Table 1.** Some programmes in Vietnam supporting AMR containment

1.	National Program for Surveillance in AMR (NPSAR) (1988 to 2006)—implemented by MoH—was a national surveillance programme for AMR. Bacteria causing infectious diseases in inpatients and outpatients were isolated and tested for antibiotic susceptibility.^11^
2.	Law on Pharmacy in 2005, Drug Law (34/2005/QH11), which is no longer valid. Applicable now is the 2016 Drug Law (law numbered 105/2016/QH13 by the National Assembly). This law prohibits selling prescription drugs to anyone without a prescription.^12^
3.	In June 2015, the Aide Memoire on ‘Multi-stakeholder Engagement to combat AMR in Viet Nam’ was jointly signed by the MoH, MARD, Ministry of Industry and Trade and the Ministry of Natural Resources and Environment, including other development partners such as WHO, FAO and OUCRU.^7^
4.	The Vietnam resistance project (VINARES) was an AMR surveillance network established in 2012. This network was recognized as the national AMR surveillance network in 2016 by the MoH and includes 16 central and provincial hospitals.^2^
5.	Annual communication activity/meeting on AMU and AMR in November.^13^
6.	Local antibiotic prescribing guidelines by MoH as treatment guidelines and antimicrobial stewardship guidelines.^14^
7.	In 2016, to support a multisectoral approach, the MoH established the National Steering Committee on Prevention of AMR for 2016–2020.^10^
8.	The NAP on Infection Control of the Health Sector for the period 2016–2020.^15^
9.	In 2020, MARD issued a Circular, which provides the list of antibiotics and timelines to phase out the preventive use of antibiotics in animal husbandry.^16^

This list is not exhaustive and outlines only few of the programmes.

The multilayered nature of AMR requires stakeholder involvement across different ‘One Health’ sectors. This cross-sectoral approach needs to be supplemented by an understanding of the community-level drivers of AMU. The UN’s Inter-Agency Coordination Group on AMR, in its 2019 report to the UN Secretary-General, has highlighted the need to engage community stakeholder groups and to increase community ownership for AMR mitigation efforts.^17^

Despite the understanding that public engagement is key to the success of any multisectoral programme or policy implementation, only a few NAPs on AMR have developed this narrative. An analysis of NAPs in the SEA region showed that most countries did not prioritize stakeholder participation or community engagement.^18^ Even minimal public engagement mentioned in some NAPs envisaged a top-down approach. Although several European countries have experience of AMR programmes for the past two decades, citizen and stakeholder roles have not been defined in their NAPs. Keeping these points in mind, we attempted to explore the understanding of AMR and its drivers among various stakeholder groups while simultaneously analysing public participation in Vietnam’s NAP implementation efforts.

## Methods

A qualitative exploratory approach was used as the drivers of AMR are often complex and multisectoral (at the community level). KIIs were used as the methodology for data collection, and the study period was from August–October 2021 with the study setting defined as the country of Vietnam. An interview tool was prepared and validated based on a consultative process involving researchers from Vietnam and India. The tool looked at different themes on awareness of antibiotic use and antibiotic resistance, Vietnam’s NAP for AMR and its impact, programmes aimed at rationalizing AMU in Vietnam, and the perceptions towards the successful implementation of NAPs with robust community participation (the interview tool is presented as Annex 1, available as Supplementary data at *JAC-AMR* Online).

Purposive sampling was used to identify the study participants. Participants were first contacted by telephone. Key informants were identified as those who were engaged with a substantial scale of AMU, diagnosis of infections or concerned with antimicrobial content in effluents in their professional life. They also had to have significant experience working with communities or grassroots-level institutions. Participants were selected from the human, animal and plant health sectors to gain a multidimensional perspective of the ground-level situation. Thirteen members were approached and all except one, who claimed a conflict of interest with the pharmaceutical company that employed him, consented to participate in the study. Six participants each from northern and southern Vietnam were selected to ensure equal representation.

Table 2 provides basic information about study participants while keeping their identity confidential. Verbal informed consent was obtained in recorded format after explaining the purpose and process of the study. The interviews were structured according to the qualitative interview tool, conducted in the language most comfortable for the participants (either Vietnamese or English) and recorded utilizing Zoom Meetings (a proprietary videotelephony software program developed by Zoom Video Communications or a voice recorder device). The participants were compensated for the time they spent for this study. The average duration of the interview was 45–60 min.

**Table 2.** Participant details

Study participant code	Sector/domain	Informant profile—working relation with AMU/AMR	Gender/age (years)	Years of experience
H1	Human	Pharmacist, self-owned pharmacy	F/31	5
H2	Human	Infectious diseases doctor at a tertiary care hospital	F/35	10
H3	Human	Hospital laboratory technician at a referral hospital under Department of Health	F/33	11
H4	Human	Lecturer of Pharmaceutical Economic Management at university	F/39	15
H5	Human	Primary care doctor, Head of Commune Health Station	M/50	21
H6	Human	Pharmaceutical sales representative, private sector	M/40	24
A1	Animal	Swine farmer	M/32	7
A2	Animal	Researcher on AMR and AMU working on government projects	M/37	10
A3	Animal	Veterinary health worker, private clinic	M/35	13
A4	Animal	Veterinary drug store owner at sub-department of animal health	M/41	17
A5	Animal	Aquaculture expert farmer	M/35	17
E1	Environment	Lecturer of Biotechnology at University of Agriculture and Forestry	M/45	19

M, male; F, female.

Two female researchers based in Vietnam with a good understanding of the AMR landscape and qualitative study methods conducted the interviews. The interviews were later transcribed verbatim and translated into English by the researchers. The validity of the translation was ascertained independently. The transcripts were not shared with the informants.

The data were entered into Microsoft Excel, and manifest and latent content analysis was done. The codes were categorized based on the study objectives, and categories were developed into themes based on the preformed understanding of the study topic. Coding and analysis were verified and validated at multiple stages by two researchers in the team independently.

### Ethics`

Ethics committee approval was obtained through the Institutional Ethics Committee, Pushpagiri Institute of Medical Sciences and Research Centre, Thiruvalla—Kerala, India under the IRB Study Reference No: 08/08/2022.

## Results

**Figure 1.**
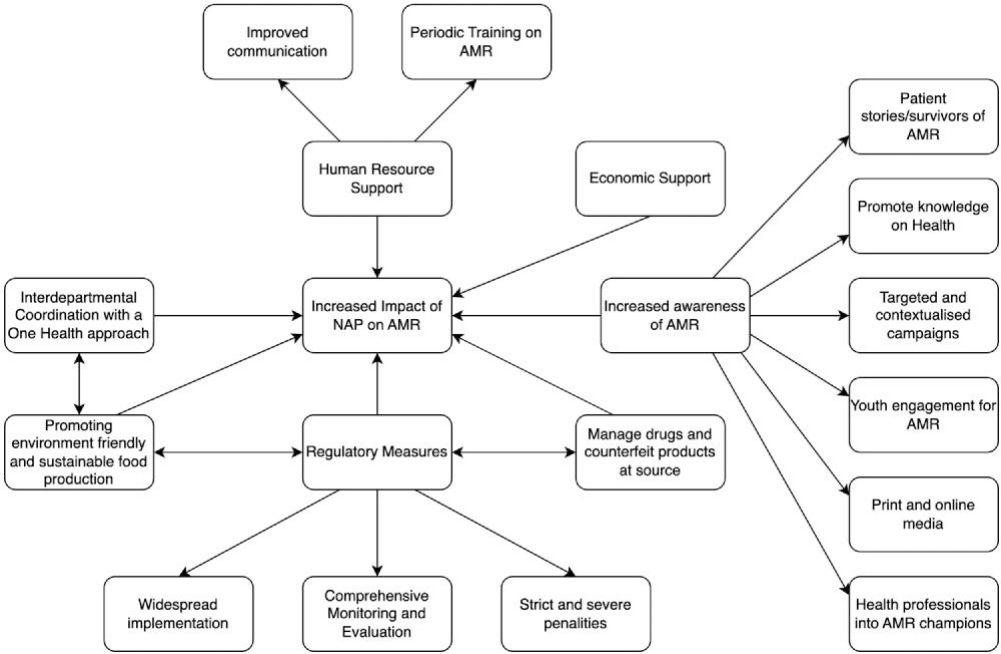
Concept map 1. Methods to increase the community-level ownership and impact of NAPs on AMR.

In exploring the methods to increase community-level ownership and the impact of NAP on AMR, the study participants had a selection of responses, which have been summarized in Concept Map 1 (Figure 1) in a concise format. It was found that increasing the awareness on AMR through multiple measures such as targeted and contextualized campaigns, print and online media along with widespread implementation, comprehensive monitoring and evaluation of regulatory measures were some of the measures for increasing the impact of NAP on AMR.

The demographic and occupational profiles of the study participants are given in Table 2. The analysis of the KIIs revealed the following highlights based on the themes.

### General awareness of AMR

Participants reported awareness of programmes on AMR conducted by hospitals and universities. It was observed that they had a broad grasp of AMR and its effects, as well as a caution against self-medication due to the belief that drugs are harmful to the body if taken regularly.

*‘I take extra precautions to reduce my use of antibiotics when I travel internationally. Even if I get a fever, I don't take antibiotics unless I have a prescription. A wrong dosage will contribute to drug resistance, which will negatively impact human health.’—E1*

At the same time, most participants did not consider AMR as receiving a high priority from the government or the public. Pertaining to the human health sector, one participant remarked on the poor awareness of AMR among the public and another on the difficulty in translating knowledge on AMR to actions or behaviours. In the lives of farmers, it was reported that infectious diseases had more visible consequences than AMR, and awareness of AMR was lacking.

‘*The government has conducted multiple public engagement activities on AMR, but they do not seem to successfully create changes in public behaviour. My mother still buys antibiotics without a prescription, and my colleagues still use antibiotics in high doses. I think the National Action Plan does not reach nor create an effect in the community.’—H3*

It was found that MARD has a list of permitted medicine products each year. Sub-Departments of Animal Health and Livestock Production (SDAH-LP) at provincial levels introduce this list to pharmacy drug stores, and farmers can then access this information from the pharmacists. The SDAH-LP in certain provinces provides training biannually on animal husbandry, disease prevention, new diseases, and new drugs to increase the general awareness of AMR.

### Capacity building and quality assurance initiatives

The participants mentioned multiple measures that increased the capacity of the healthcare delivery system to tackle the various drivers of AMR. It was noticed that pharmacists provided instructions on how to use antibiotics for human health with every purchase that was made. The government has regulations stipulating that pharmacists must hold a university pharmacy degree and that those selling antibiotics must obtain a certificate to update their professional knowledge every 3 years.

*‘There is the easy availability of antibiotics for purchase. However, the pharmacists provide instructions for usage and some advice such as: ‘this is the strongest antibiotic, just use for 3 days’ or ‘these are a critical class of antibiotics drugs, please use normal class’. I have significantly reduced my usage of antibiotics.’—H3*

Initiatives from the MoH and Drug Management Office, such as the collection of expired medicines from pharmacies at commune health centres (CHCs) and their disposal routed through a District Health Centre (DHC), has reduced some amount of environmental contamination and aided in the AMR mitigation efforts. The DHC handles the quantity and quality of drugs and organizes training for healthcare workers in CHCs.

Increased knowledge about AMR among professionals of human health, animal health and agriculture was reported. Some healthcare facilities have also launched antimicrobial stewardship programmes with an active audit of prescriptions.

*‘The SDAH invites me to attend the training on animal husbandry and disease prevention twice a year. They also provide training on new decrees released as well as new drugs.’—A5*

The lab capacity for doing antibiotic sensitivity testing and systems for compiling the results have significantly increased since the action plan’s launch by MoH, pertaining to the human health sector. Under the Direction of Healthcare Activities (DOHA) scheme launched in 2010, there have been improvements to the healthcare delivery facilities, particularly at the level of districts and communes.

### Guidelines and regulations for AMR containment

In an ongoing study being done by one of the participants, it was found that antibiotic usage in food animals has reduced compared with the previous 4 years. There are also regulations on antibiotics that cannot be used in animals or antibiotic withdrawal time before slaughter. These findings are specified by the participant as localized and attributable mostly to technical and NGO-funded interventions targeting antibiotic reduction.

*‘In 2020, the government banned antibiotics for growth promotion in animal husbandry. My ongoing studies show that the usage of antibiotics has reduced in comparison to 4 years earlier. All antibiotics that affect human health have been banned for use in the animal sector.’—A2*

The NAP is integrated into the annual plan for animal disease prevention programmes and the SDAH staff receive adequate AMR training; but farmers and the general public are often not sensitized to the same level. Besides, there is an underdeveloped lab capacity for testing with an inadequate collection of data on AMU and AMR in the farm sector.

Antibiotics’ monitoring bodies are present for human and animal health sectors, led by the MoH and the MARD, respectively. The authorities inspect stores selling antibiotics three to four times a year. Authorities make use of digital management, market surveillance agencies, district-level interdepartmental agencies, and agricultural department inspectors, among other resources, for monitoring purposes.

Specifically, for the veterinary sector, the SDAH-LP in each province issues a certificate of eligibility to sell drugs. Veterinary authorities at the province and district levels can examine and take the drug samples and impose penalties on pharmacists.

Water, hygiene and sanitation practices have improved considerably in communities, farms and hospitals in the past 10 years due to better economic conditions and guidelines/campaigns by the government. Government agencies have provided several guidelines on hygiene and water treatments on farms. Since the New Rural Development campaign, which started nationwide in 2010, people have better understood hygiene and the need to keep the environment clean.

Hygiene improvements in healthcare facilities were affected through improved infrastructure with a focus on building better service delivery. Besides, the Infection Prevention & Control (IPC) practices also improved considerably in healthcare facilities through a NAP on IPC (2016–20).

*‘I had been in Obstetrics and Gynaecology in a hospital in Hanoi around 7 years ago. At that time, the treatment area had only one shared toilet for patients and hospital staff. But now, with increased funding support, the hospital has been rebuilt and upgraded with separate rooms and toilets.’—H4*

### Suboptimal accountability in prescribing and sales of antibiotics

The findings from KIIs state that by law, each pharmacy must have a computer connected to the internet with software installed, mandated by the MoH. Sales data must be entered into the software. However, the government is unable to enforce it efficiently because of challenges in regulating many retail pharmacies in the country.

With reference to animal health, the SDAH-LP in some provinces recommend treatment regimens, training for veterinarians, improving knowledge about AMR, and increasing the competence to diagnose and treat diseases. Similarly, the MoH provides treatment pathways for medical practitioners to follow. However, these regulatory mechanisms and guideline documents on appropriate antibiotic use are not effectively enforced due to capacity challenges across sectors.

*‘Antibiotic prescribing has not been reduced. Doctors always prescribe antibiotics in all prescriptions. If not one type, then another.’—H6*

*‘The situation has not changed much; farmers can easily buy antibiotic-containing products without a prescription.’—A4*

## Discussion

The 2021 Global Antimicrobial Resistance and Use Surveillance System report by the WHO has emphasized the need for robust action for AMR containment in the SEA Region.^19^ In Vietnam, an increase in the use of antibiotics was noted between 2000 and 2018 (from 6.3 DDD/1000/day to 30 DDD/1000/day).^20^

Many AMR studies from Vietnam are limited to individual sectors with some analysis contextualizing the subject through an SEA perspective or sector-wise impact on animal health and human health, challenges in AMR surveillance, governance and other themes.^21,22^

The present study is unique in its approach that it does not limit itself to any sector and therefore brings out a holistic overview of the public perception and community-level effects of the NAPs on AMR in Vietnam.

Authors in this study found that awareness about antibiotic use and AMR was adequate among study participants. However, their knowledge on the NAP on AMR and its full mandate was less than optimal even though most were engaged with AMR-curbing activities. This emphasizes the need for more sensitization with a focus on linkages of antibiotic use and its implications across sectors. Though the ‘One Health’ approach is incorporated into most NAPs across LMICs, actual cross-sectoral coordination is a challenge during implementation.^23^ The authors feel that a lack of adequate governance mechanisms in the form of a dedicated secretariat and dedicated staff further compounds this challenge.

Our findings agree with other studies, where a lack of awareness of AMR among the public is being noted. Though the involvement of communities is increasingly promoted for tackling AMR, there are no proven models yet.^24^ Besides, due to varied socioeconomic and cultural practices, different communities may need different components based on their culture and context. There is a definite need to prioritize this issue and improve awareness among different community sections with straightforward messages through campaigns. The involvement of village or province leaders for consultations in the design and rollout of AMR policies will be a good start. Though there are other competing priorities, explaining interlinkages between AMR and its drivers (hygiene and infection prevention), and its potential immediate benefits—on preventing infections, avoiding loss of income from sickness, impact on food quality, food security and environment contamination, all affecting the long-term social development of future generations—may help in mobilizing resources and prioritizing AMR.

Though there were many significant changes (including key policy norms) post the launch of the NAP on AMR in Vietnam, not all of these could be directly attributed to NAP implementation efforts. Improved surveillance efforts could be partly attributed to the Fleming fund.^25^

Unless supplemented by educating the public on the need for appropriate AMU and the risks associated with AMR, all regulatory measures may have shortfalls in their effectiveness. However, the transition from maximizing short-term economic benefits to long-term AMR prevention must happen without impacting the livelihoods of those depending on these measures, especially from the farm/animal sector. As stated by the study participants, suboptimal adherence to regulations pertaining to over-the-counter sale and usage of antibiotics is a problem not only in Vietnam but also in other LMICs.^26^

Supply of antibiotics in the animal sector is a profitable and competitive business in Vietnam. Perverse incentives may be available at various antibiotic value-chain levels to increase the use of antibiotics in animal health and food production. The farmers also find the use of antibiotics profitable with immediate results. Therefore, the economic drivers of antibiotic use in community settings should be factored in while strategizing the success of NAP implementation efforts at ground level. In the human health sector, economic losses for business owners (chemists’ outlet) have limited the impact of attempts to address the inappropriate use and sale of antibiotics.

A study based in SEA also states that a suboptimal understanding of antimicrobials magnifies the risk of self-medication, unregulated access, irrational prescription, and other failures of compliance with regulatory measures.^27^

The AMR-related capacity building over the last decade lacks alignment with the NAP and its regulatory efforts. Though successful in its scope, it has multiple challenges. There is a need to complement regulatory measures with public education on AMR and contextualize guidance on the effects of AMR at ground level.^28^

There is a need for NAPs to be sensitive to the structural challenges faced by LMICs, including the lack of an investment case to prioritize AMR action at various levels. LMICs not only suffer from problems such as lack of access to clean water and sanitation but also suffer from the AMR burden. Therefore, sustained support is essential for AMR mitigation efforts.^29^

There is a low priority given to community components of NAP, and there is a need to improve community ownership to induce behaviour changes over time towards more appropriate use of antibiotics and public support of interventions to tackle AMR. At the primary and community level of care in Vietnam, antibiotic use is driven by the community members’ social and economic response to what constitutes effective healthcare rather than biomedical logic.^27^ Therefore, engaging with community values and structural conditions that drive antibiotic use is necessary. Equally important is to tailor interventions with alternatives that provide similar perceived benefits to antibiotics such that there is a reduction in widespread antibiotic misuse.

There is a need to revitalize the implementation machinery of the NAP on AMR in Vietnam. Improving the enforcement capacity of regulations and targeting community ownership is essential for the success of the NAP 2.0 and obtain sustainable change on the ground.

## Study limitations

The study attempted to capture the impact of the NAP on AMR in Vietnam with a ‘One Health’ lens but is limited in its scope of the community perspective, as all study participants were professionals handling antibiotics in their daily work.

### Conclusions

The country has made good progress in moving forward with various components in the NAP. However, for the successful implementation of NAP 2.0, targeting community-level ownership and increased public AMR awareness through localized and contextualized efforts should be considered. It is recommended that increasing human resource support and economic support, alongside strengthening regulatory measures for managing drugs and counterfeit produce at source, will further improve implementation measures. Promoting interdepartmental coordination with a One Health approach and environment-friendly and sustainable food production will also be important. It is hoped that the perspectives from this study and recommendation will greatly help in laying the platform for augmenting the impact of NAP on AMR in Vietnam.

## Supplementary Material

dlad146_Supplementary_Data

## Supplementary data

Annex 1 is available as Supplementary data at *JAC-AMR* Online.
