# Supplementary material for: Public perception and community-level impact of national action plans on antimicrobial resistance in Vietnam
Source: JAC Antimicrob Resist. 2023 Dec 28;6(1):dlad146. doi: 10.1093/jacamr/dlad146 (PMC10753920; doi:10.1093/jacamr/dlad146)
Supplement: dlad146_Supplementary_Data [file dlad146_supplementary_data.docx]

**Supplementary Data**

**Annex 1**

Qualitative Study Tool

1. Introduction

● Introduction to researcher

● Study topic

● Rationale behind the study

● Explanation of the objectives of the study

● Explain confidentiality and anonymity

● Explain recording, length, and nature of the discussion.

● Seek consent.

● Ask participants for permission to archive the transcript of the interview. Explain that it will be fully anonymized and allow other researchers to use the information they have given for research purposes.

● Check whether they have any questions and get a consent signed

2. Participant Introduction:

● Ask the participant for a self-introduction.

● Name, Age, Educational background

3. Occupational Background:

● Current line of occupation/profession with different job roles, if any held to date.

● Years of work experience

● Ask whether their line of work involves healthcare or dealing with antibiotics in any form

4. Antibiotic Use

● Describe the use of antibiotics in your occupation. Reason for use, Mode of procurement, method of use, and Mode of disposal

○ If they use antibiotics, ask them about any guidelines they follow in selecting the type and quantity

○ Any changes over the last decade

● Describe the difference in your work in the initial phases of your profession in comparison to current in the context of antibiotic use.

○ Ask about the commonest antibiotics used earlier and now, if the interviewee is a prescriber

○ On average, how many patients/sick-animals will be receiving antibiotics from the health/vet centre, any changes over time?

○ Ask about improvements in infection prevention practices- Water, Sanitation & Hygiene (WASH) in communities and Infection Prevention & Control (IPC) in healthcare settings

○ If the interviewee is a farmer or veterinarian, ask about antibiotic use in food animal production and possible changes over time in terms of growth promotion, prophylaxis, and treatment.

○ If the interviewee is a non-prescriber, enquire about the differences in antibiotic procurement, use or disposal in their line of occupation before 7-8 years and now.

● Are you aware of any regulatory/monitoring body which enquires/regulates the way in which antibiotics are used in your sector? (use of antibiotics and exposure to harmful antibiotics)

○ If they are aware, ask them about the process of monitoring or regulatory oversight (e.g. audits, reporting etc.)

○ How frequent and effective is the oversight

○ Any changes over the last one decade

● In your opinion, which sector misuses antibiotics the most and what can be done about it?

● Can antibiotics be procured easily, without professional advice, in your country in any sector? Are they procured as raw antibiotics or registered?

5. Knowledge about Antibiotic Resistance

● Have you heard of the term antibiotic/antimicrobial resistance?

○ If yes, what was the context?

○ Ask about the factors which promotes resistance

○ Follow Up with questions on their perception of the importance of the problem of ABR/AMR

○ If they are familiar with the term antibiotics, ask about their opinion on calling it as a finite natural resource

● Have you taken part in any government/ public or private organization awareness programs regarding antibiotics or AMR?

○ If yes, ask them whether they can describe the content of those programs and the take home messages

○ Did those programs benefit the interviewee and other participants?

● Do you know of any disadvantages/ill effects of inappropriate antibiotic use? How have you come about the information? (Year and occasion/event/media)

○ Ask about how we can categorize antibiotic use as appropriate and inappropriate

● If they know about some disadvantages of inappropriate antibiotic use, ask them about ways to prevent it

6. National Action Plan on Antimicrobial Resistance

● Have you heard about the National Action Plan on AMR?

○ If yes, what was the context?

○ If yes, ask about the functionality and impact of the National Action Plan on AMR

○ Has the NAP been successful in changing things on the ground?

○ Has the pandemic situation affected the NAP in any way?

● Are there any rules/regulations/programs that came in the wake of NAP that has affected the use of antibiotics or overall WASH/IPC in any way?

○ If yes, ask about each dimension of AMR- healthcare, agriculture, and environment

● Do you feel that the mandate of NAP should be renewed or extended?

○ If yes, what would you like to see in NAP 2.0?

○ How can we improve the impact of the National Action Plan?

○ Do you know of any successful National Action Plan for any other health or development issue we can use as a template?

7. Way forward

● Thoughts on how ABR can be contained by interventions. (Both private and public)

● How can the general awareness about antibiotic misuse and ABR go up among various stakeholder groups?

● How do we identify ‘low hanging fruits’ for AMR action? (The question on where to start)

● How can we increase the political capital of the issue or how can we influence the policy community about prioritizing ABR?

● Are there any AMR champions that we can include in our work?

● If relevant to the professional background of the interviewee, ask about how to mobilize finances for implementing NAPs

8. Conclusion

● Thank the participants for their time.

● Check if they have any questions before concluding.
